# Comparison of the Ability of Different Clinical Treatment Scores to Estimate Prognosis in High-Risk Early Breast Cancer Patients: A Hellenic Cooperative Oncology Group Study

**DOI:** 10.1371/journal.pone.0164013

**Published:** 2016-10-03

**Authors:** Flora Stavridi, Konstantine T. Kalogeras, Kyriaki Pliarchopoulou, Ralph M. Wirtz, Zoi Alexopoulou, Flora Zagouri, Elke Veltrup, Eleni Timotheadou, Helen Gogas, Angelos Koutras, Georgios Lazaridis, Christos Christodoulou, George Pentheroudakis, Apostolos Laskarakis, Petroula Arapantoni-Dadioti, Anna Batistatou, Maria Sotiropoulou, Gerasimos Aravantinos, Pavlos Papakostas, Paris Kosmidis, Dimitrios Pectasides, George Fountzilas

**Affiliations:** 1 Third Department of Medical Oncology, “Hygeia” Hospital, Athens, Greece; 2 Laboratory of Molecular Oncology, Hellenic Foundation for Cancer Research/Aristotle University of Thessaloniki, Thessaloniki, Greece; 3 Translational Research Section, Hellenic Cooperative Oncology Group, Data Office, Athens, Greece; 4 Oncology Section, Second Department of Internal Medicine, “Hippokration” Hospital, Athens, Greece; 5 STRATIFYER Molecular Pathology GmbH, Cologne, Germany; 6 Department of Biostatistics, Health Data Specialists Ltd, Athens, Greece; 7 Department of Clinical Therapeutics, “Alexandra” Hospital, National and Kapodistrian University of Athens School of Medicine, Athens, Greece; 8 Department of Medical Oncology, “Papageorgiou” Hospital, Aristotle University of Thessaloniki, School of Health Sciences, Faculty of Medicine, Thessaloniki, Greece; 9 First Department of Medicine, “Laiko” General Hospital, National and Kapodistrian University of Athens School of Medicine, Athens, Greece; 10 Division of Oncology, Department of Medicine, University Hospital, University of Patras Medical School, Patras, Greece; 11 Second Department of Medical Oncology, “Metropolitan” Hospital, Piraeus, Greece; 12 Department of Medical Oncology, Ioannina University Hospital, Ioannina, Greece; 13 First Department of Medical Oncology, “Metropolitan” Hospital, Piraeus, Greece; 14 Department of Pathology, Micromedica Labs, Athens, Greece; 15 Department of Pathology, Ioannina University Hospital, Ioannina, Greece; 16 Department of Pathology, “Alexandra” Hospital, Athens, Greece; 17 Second Department of Medical Oncology, “Agii Anargiri” Cancer Hospital, Athens, Greece; 18 Oncology Unit, “Hippokration” Hospital, Athens, Greece; 19 Second Department of Medical Oncology, “Hygeia” Hospital, Athens, Greece; 20 Aristotle University of Thessaloniki, Thessaloniki, Greece; University of North Carolina at Chapel Hill School of Medicine, UNITED STATES

## Abstract

**Background-Aim:**

Early breast cancer is a heterogeneous disease, and, therefore, prognostic tools have been developed to evaluate the risk for distant recurrence. In the present study, we sought to develop a risk for recurrence score (RRS) based on mRNA expression of three proliferation markers in high-risk early breast cancer patients and evaluate its ability to predict risk for relapse and death. In addition the Adjuvant! Online score (AOS) was also determined for each patient, providing a 10-year estimate of relapse and mortality risk. We then evaluated whether RRS or AOS might possibly improve the prognostic information of the clinical treatment score (CTS), a model derived from clinicopathological variables.

**Methods:**

A total of 1,681 patients, enrolled in two prospective phase III trials, were treated with anthracycline-based adjuvant chemotherapy. Sufficient RNA was extracted from 875 samples followed by multiplex quantitative reverse transcription-polymerase chain reaction for assessing RACGAP1, TOP2A and Ki67 mRNA expression. The CTS, slightly modified to fit our cohort, integrated the prognostic information from age, nodal status, tumor size, histological grade and treatment. Patients were also classified to breast cancer subtypes defined by immunohistochemistry. Likelihood ratio (LR) tests and concordance indices were used to estimate the relative increase in the amount of information provided when either RRS or AOS is added to CTS.

**Results:**

The optimal RRS, in terms of disease-free survival (DFS) and overall survival (OS), was based on the co-expression of two of the three evaluated genes (RACGAP1 and TOP2A). CTS was prognostic for DFS (p<0.001), while CTS, AOS and RRS were all prognostic for OS (p<0.001, p<0.001 and p = 0.036, respectively). The use of AOS in addition to CTS added prognostic information regarding DFS (LR-Δχ^2^ 8.7, p = 0.003), however the use of RRS in addition to CTS did not. For estimating OS, the use of either AOS or RRS in addition to CTS added significant prognostic information. Specifically, the use of both CTS and AOS had significantly better prognostic value vs. CTS alone (LR-Δχ^2^ 20.8, p<0.001), as well as the use of CTS and RRS vs. CTS alone (LR-Δχ^2^ 4.8, p = 0.028). Additionally, more patients were scored as high-risk by AOS than CTS. According to immunohistochemical subtypes, prognosis was improved in the Luminal A (LR-Δχ^2^ 7.2, p = 0.007) and Luminal B (LR-Δχ^2^ 8.3, p = 0.004) subtypes, in HER2-negative patients (LR-Δχ^2^ 23.4, p<0.001) and in patients with >3 positive nodes (LR-Δχ^2^ 23.9, p<0.001) when AOS was added to CTS.

**Conclusions:**

The current study has shown a clear benefit in predicting overall survival of high-risk early breast cancer patients when combining CTS with either AOS or RRS. The combination of CTS and AOS adds significant prognostic information compared to CTS alone for DFS, while the combination of CTS with either AOS or RRS has better prognostic value than CTS alone for OS. These findings could possibly add on the information needed for the best risk prediction strategy in high-risk early breast cancer patients in a rather simple and inexpensive way, especially in Luminal A and B subtypes, HER2-negative patients and those with >3 positive nodes.

## Introduction

Breast cancer is a heterogeneous disease with a rising incidence over recent years, with the use of adjuvant chemotherapy playing a critical role in preventing recurrence in high-risk patients. Differentiating high-risk from low-risk patients remains a critical priority and, therefore, prognostic tools have been developed to identify high-risk patients. First generation prognostic signatures, such as Oncotype Dx, Mammaprint and Prediction Analysis of Microarray 50 (PAM50), assign patients into good and poor prognosis groups based on proliferation-related genes, providing independent prognostic information complementary to standard clinicopathological parameters [1]. For example, the Oncotype DX signature was developed in order to estimate the risk for distant recurrence, while PAM50 provides an approach that identifies intrinsic subtypes [2, 3]. Also, gene expression profiling has led to new molecular classifications of breast cancer and has been developed to aid clinicians tailor therapy [4, 5]. Although first generation signatures augment the ability to select patients, this isn’t without a significant cost for health care systems.

The Oncotype DX 21-gene recurrence score (RS) was developed in a cohort of node-negative, tamoxifen-treated patients that received no cytotoxic chemotherapy and was validated in a similar independent population. RS was shown to have prognostic and predictive value in node-positive, estrogen receptor (ER)-positive postmenopausal patients treated with anastrοzole or tamoxifen in a sub-cohort of the ATAC trial [6]. Also, the 50-gene PAM50 test was developed to discriminate breast cancer subtypes (Luminal A, Luminal B, HER2-Enriched and basal like) and generated a risk of recurrence score (ROR). This score was used to identify high- and low-risk groups in node-negative and node-positive, tamoxifen-treated postmenopausal breast cancer patients [7]. The ability of the above scores to add prognostic information in ER-positive primary breast cancer patients treated with anastrozole or tamoxifen, beyond that assessed by classical clinical and immunohistochemical markers, using the clinical treatment score (CTS), was evaluated in a recent study by Dowsett et al. [8]. CTS integrated prognostic information from age, nodal status, tumor size, histological grade and treatment. The study showed that ROR provides more prognostic information in ER-positive, node-negative patients, compared to RS and differentiates more efficiently intermediate and high-risk patients.

It has been shown that proliferation is the strongest parameter predicting clinical outcome in hormone receptor-positive, HER2-negative patients, while immune response and tumor invasion appear to be the main biological processes involved in triple-negative and HER2-positive breast cancer. However, the use of over-complex methods does not appear to result in improvement of the accuracy of prediction. Furthermore, prognostication models using small sets of selected genes have shown similar or even better performance than models fitted from genome-wide data. Zhao et al. [9] investigated the prognostic/predictive value of 9 expression-based gene signatures, including PAM50, Oncotype DX and genomic grade index, in a cohort of 947 patients included in six published breast cancer microarray datasets. The signatures were able to predict recurrence within the first 5 years, presumably because they picked-up cases with the most aggressive biology, since they access proliferation. Therefore, models using a small set of selected biologically driven genes are cheaper and friendlier and appear to be more effective in predicting risk [10].

Since proliferation markers appear to play a significant prognostic/predictive role, the use of two or three markers instead of multi gene signatures, might be sufficient to predict risk for recurrence. Three important proliferation genes for breast cancer are RACGAP1, TOP2A and Ki67. It has been shown that the *RACGAP1* gene encodes Rac GTPase activating protein 1 (RACGAP1), which plays a role in cell proliferation, motility, invasion and metastasis of breast cancer cells [11–14]. In a study of high-risk breast cancer patients treated with postoperative dose-dense chemotherapy, it was shown that high RACGAP1 mRNA expression was of adverse prognostic significance in terms of DFS and OS [15]. Also, the topoisomerase II alpha (*TOP2A*) gene encodes the alpha isozyme of topoisomerase II, which is responsible for transcription, replication and chromosome condensation and segregation during cell division [16]. It is amplified in 30–60% of the tumors with *HER2* amplification [17]. Additionally, recently, there has been interest in assessing Ki67 as a prognostic/predictive marker, as it has been shown to correlate with grade, while it is recommended to be evaluated along with traditional parameters, when choosing adjuvant therapies [18].

Adjuvant! Online for breast cancer is an open access computer program designed to assist physicians in making decisions regarding adjuvant therapy in patients with early breast cancer by predicting risk for relapse and individual 10-year survival probabilities [19]. The program has been validated by several groups in Europe, Asia and Canada with contradictory results [20–23].

Adjuvant! Online is a useful tool, it has however its limitations. In a cohort of 1,065 British breast cancer patients Adjuvant! Online was found to significantly overestimate overall survival and breast cancer-specific and event-free survival. This overestimation of breast cancer-specific survival underestimated breast cancer mortality in the UK [20]. In another Dutch study, Adjuvant! Online overestimated overall survival in the subgroups of patients <40 years and >69 years of age, while it underestimated breast cancer-specific survival in the patient subgroup of 1–3 positive lymph nodes [21]. Similarly, van Kleef et al. evaluating, in a retrospective manner, a large series of Dutch patients with early breast cancer also concluded that Adjuvant! Online underestimates both 10-year overall survival, as well as breast cancer-specific survival [24] and that it should be used in clinical practice with caution.

In the present study, we sought to develop a risk for recurrence score (RRS) based on three proliferation markers, e.g. RACGAP1, TOP2A and Ki67, in high-risk early breast cancer patients and evaluate its ability to predict risk for relapse and death. In addition the Adjuvant! Online score (AOS) was also determined for each patient, providing a 10-year estimate of relapse and mortality risk. We then evaluated whether RRS and/or AOS might possibly improve the prognostic information of a slightly modified version of the CTS, in much the same way Dowsett et al. [8] evaluated the ability of RS and ROR to improve the prognostic information of the CTS.

## Materials and Methods

### Patient population

This was a retrospective translational research study among 1,681 high-risk early breast cancer patients, enrolled in two prospective phase III trials. The HE10/97 trial [25] was a randomized phase III trial (ACTRN12611000506998) in patients with high-risk node-negative or intermediate/high-risk node-positive operable breast cancer, comparing four cycles of epirubicin (E) followed by four cycles of intensified CMF (E-CMF) with three cycles of E, followed by three cycles of paclitaxel (T, Taxol®, Bristol Myers-Squibb, Princeton, NJ) followed by three cycles of intensified CMF (E-T-CMF). All cycles were given every two weeks with G-CSF support. Dose intensity of all drugs in both treatment arms was identical, but cumulative doses and duration of chemotherapy period differed. In total, 595 eligible patients entered the study in a period of 3.5 years (1997–2000).

The HE10/00 trial [26, 27] was a randomized phase III trial (ACTRN12609001036202), in which patients were treated with E-T-CMF (exactly as in the HE10/97 trial) or with four cycles of epirubicin/paclitaxel (ET) combination (given on the same day) every three weeks followed by three cycles of intensified CMF every two weeks (ET-CMF). By study design, the cumulative doses and the chemotherapy duration were identical in the two arms but dose intensity of epirubicin and paclitaxel was double in the E-T-CMF arm. A total of 1,086 eligible patients with node-positive operable breast cancer were accrued in a period of 5 years (2000–2005).

HER2-positive patients received trastuzumab upon relapse, as previously described [28]. Treatment schedules for the two studies are shown in S1 Table. Baseline characteristics and clinical outcomes of both trials have already been described in detail [25–27, 29]. Primary tumor diameter, axillary nodal status and tumor grade were obtained from the pathology report. Clinical protocols were approved by local regulatory authorities, while the present translational research study was approved by the “Papageorgiou” Hospital Institutional Review Board (July 15, 2013) and the Bioethics Committee of the Aristotle University of Thessaloniki School of Medicine (December 18, 2013). All patients signed a study-specific written informed consent before randomization, which in addition to giving consent for the trial allowed the use of biological material for future research purposes. All clinical investigations related to the present study have been conducted according to the principles expressed in the Declaration of Helsinki.

### Tissue microarray (TMA) construction

**Fig 1:**
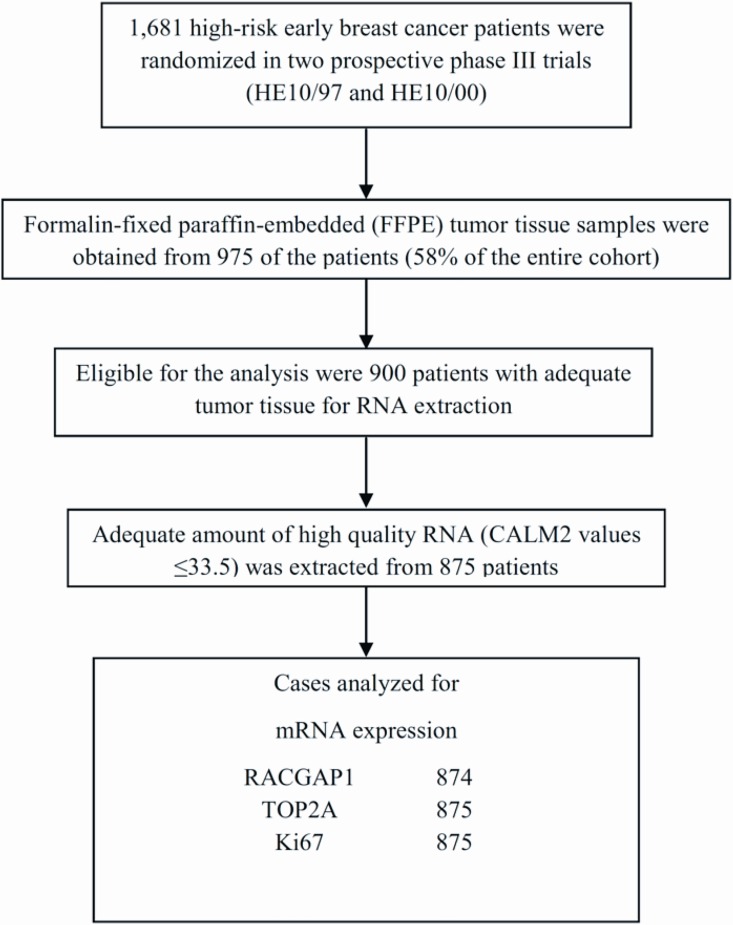
REMARK diagram.

Formalin-fixed paraffin-embedded (FFPE) tumor tissue samples from 975 patients (58.0% of 1,681 randomized patients) were collected from both trials, retrospectively in the first (HE10/97) and prospectively in the second (HE10/00). Of these, 900 (92.3%) had enough material left for RNA isolation needed for the present study. The REMARK diagram [30] for the study is shown in Fig 1. Hematoxylin-eosin stained sections from the tissue blocks were reviewed by two experienced breast cancer pathologists and the most representative tumor areas were marked for the construction of the ΤΜΑ blocks with the use of a manual arrayer (Model I, Beecher Instruments, San Prairie, WI), as previously described [31, 32]. Each case was represented by 2 tissue cores, 1.5 mm in diameter, obtained from the most representative areas of primary invasive tumors or in some cases (9.6%) from synchronous axillary lymph node metastases and re-embedded in 51 microarray blocks. Each TMA block contained 38 to 66 tissue cores from the original tumor tissue blocks, while cores from various neoplastic, non-neoplastic and reactive tissues were also included, serving as orientation controls for slide-based assays. Cases not represented, damaged or inadequate on the TMA sections (82 of 975, 8.4%) were re-cut from the original blocks and these sections were successfully used (96%) for protein and gene analysis.

### Immunohistochemistry (IHC)

Immunohistochemical labeling was performed according to standard protocols on serial 2.5 μm thick sections from the original blocks or the TMA blocks. All cases were also stained for vimentin (clone V9, Dako, Glostrup, Denmark) and cytokeratin 8/18 (clone 5D3, Novocastra^TM^, Leica Biosystems, Newcastle, U.K), which were used as control stains for tissue immunoreactivity and fixation, as well as identification of tumor cells. Tissue samples negative for the above antibodies (21 of 975, 2.2%) were excluded from the study. To assure optimal reactivity, immunostaining was applied 7 to 10 days after sectioning at the Laboratory of Molecular Oncology of the Hellenic Foundation for Cancer Research, Aristotle University of Thessaloniki School of Medicine. The staining procedures for vimentin, cytokeratin 8/18, HER2 (A0485 polyclonal antibody, Dako), estrogen receptor (ER, clone 6F11, Novocastra^TM^, Leica Biosystems), progesterone receptor (PgR, clone 1A6, Novocastra^TM^, Leica Biosystems) and Ki67 (clone MIB-1, Dako) were performed using a Bond Max^TM^ autostainer (Leica Microsystems, Wetzlar, Germany), as previously described in detail [33–35].

### Interpretation of the IHC results

The evaluation of all IHC sections was done by two experienced breast cancer pathologists, blinded as to the patients’ clinical characteristics and survival data, according to existing established criteria, as previously described [28]. Briefly, HER2 protein expression was scored in a scale from 0 to 3+, the latter corresponding to uniform, intense membrane staining in >30% invasive tumor cells [36]; ER and PgR were evaluated using the Histoscore method (max score: 400) and were considered positive if staining was present in ≥1% of tumor cell nuclei [37]; and, for Ki67, the expression was defined as low (<14%) or high (≥14%) based on the percentage of stained/unstained nuclei from the tumor areas [38]. The mean percentage of stained cells from the two cores was calculated, while in cases with different intensities, the higher intensity score obtained from the two cores was used. If one of the tissue cores was lost or damaged the overall score was determined from the remaining one. When whole tissue sections were used, the entire tumor area was evaluated.

### Fluorescence in situ hybridization (FISH)

TMA sections or whole tissue sections (5 μm thick) were used for FISH analysis, using the ZytoLight^®^ SPEC *HER2*/*TOP2A*/CEP17 triple color probe (ZytoVision, Bremerhaven, Germany), as previously described [39]. FISH was performed according to the manufacturer’s protocol with minor modifications in all cases, not only the HER2 IHC 2+ cases. Four carcinoma cell lines (MDA-MB-231, MDA-MB-175, MDA-MB-453 and SK-BR-3) from the Oracle HER2 Control Slide (Leica Biosystems), with a known *HER2* gene status, were also used as a control for the FISH assays and analyzed for *HER2* genomic status.

For all probes, sequential (5 planes at 1.0 μm) digital images were captured using the Plan Apo VC 100x/1.40 oil objective (Nikon, Japan) using specific filters for each probe. The resulting images were reconstructed using specifically developed software for cytogenetics (XCyto-Gen, ALPHELYS, Plaisir, France). Processed sections were considered eligible for FISH evaluation according to the ASCO/CAP criteria [36]. For the evaluation of the *HER2* gene status, non-overlapping nuclei from the invasive part of the tumor were randomly selected, according to morphological criteria using DAPI staining, and scored. The virtual slides of HER2, ER or PgR stains, created as previously described [33], were used for selecting the invasive part of the tumor in each TMA. Twenty tumor nuclei were counted according to Press et al [40]. The *HER2* gene was considered to be amplified when the *HER2*/CEP17 ratio was >2.2 [36], or the mean *HER2* copy number was >6 [41]. In cases with values at or near the cut-off (1.8–2.2), 20–40 additional nuclei were counted and the ratio was recalculated. In cases with a borderline ratio, additional FISH assays were performed in whole sections [42]. The data from the evaluation of *TOP2A* gene status were neither analyzed nor presented in the present manuscript. All primary image data of the TMA and whole tumor sections have been digitally scanned and made publicly available at: https://figshare.com/articles/Photos_of_TMA_and_whole_tumor_sections/3485879

### RNA isolation and quantitative reverse transcription-polymerase chain reaction (qRT-PCR) assessment

Prior to RNA isolation, macrodissection of tumor areas was performed in most (69%) of the FFPE sections (all sections with <50% tumor cell content). More than one FFPE section (2–8 sections, 10 μm thick) was used for RNA extraction when the tumor surface of a given sample was less than 0.25 cm^2^. From each FFPE section or macrodissected tissue fragments, RNA was extracted using a standardized fully automated isolation method for total RNA from FFPE tissue, based on germanium-coated magnetic beads (XTRAKT kit, STRATIFYER Molecular Pathology GmbH, Cologne, Germany) in combination with a liquid handling robot (XTRAKT XL, STRATIFYER Molecular Pathology GmbH), as previously described in detail [34, 43, 44]. The method involves extraction-integrated deparaffinization and DNase I digestion steps. The quality and quantity of RNA was checked by measuring CALM2 expression as a surrogate for amplifiable mRNA by qRT-PCR. CALM2 was used as endogenous reference, since it had previously been identified as being highly and stably expressed among breast cancer tissue samples.

qRT-PCR primers and labeled hydrolysis probes were selected using Primer Express® Software, Version 2.2 and 3 (Applied Biosystems/Life Technologies, Karlsruhe, Germany), according to the manufacturer’s instructions, and were controlled for single nucleotide polymorphisms. All primers, probes and amplicons were checked for their specificity against nucleotide databases at NCBI using Basic Local Alignment Search Tool (BLAST). Primers and probes were purchased from Eurogentec S.A. (Seraing, Belgium). For each primer/probe set, the amplification efficiency was tested, aiming to reach comparable efficiency of >90% (efficiency range from 98 to 101%). Primers and hydrolysis probes were diluted to 100 μM, using a stock solution with nuclease-free water (Life Technologies GmbH, Darmstadt, Germany) [44]. qRT-PCR was applied for the relative quantification (RQ) of RACGAP1, TOP2A and Ki67. The Primer/Probe (ATTO/ROX-labeled) sets used for amplification of the target and reference genes are shown in Table 1.

**Table 1:** Primer and probe sequences used for quantitative reverse transcription-polymerase chain reaction (qRT-PCR).

Gene Symbol	NM_Number	Probe Name	Probe Sequence	Forward Name	Forward Sequence	Reverse Name	Reverse Sequence
**RACGAP1**	NM_013277	MP502	ACTGAGAATCTCCACCCGGCGCA	MP502_For	TCGCCAACTGGATAAATTGGA	MP502_Rev	GAATGTGCGGAATCTGTTTGAG
**TOP2A**	NM_001067	MP495	CAGATCAGGACCAAGATGGTTCCCACAT	MP495_For	CATTGAAGACGCTTCGTTATGG	MP495_Rev	CCAGTTGTGATGGATAAAATTAATCAG
**Ki67**	NM_002417	MP552	ACGGTCCCCACTTTCCCCTGAGC	MP552_For	CGAGACGCCTGGTTACTATCAA	MP552_Rev	GGATACGGATGTCACATTCAATACC
**CALM2**	NM_001743	MP501	TCGCGTCTCGGAAACCGGTAGC	MP501_For	GAGCGAGCTGAGTGGTTGTG	MP501_Rev	AGTCAGTTGGTCAGCCATGCT

For PCR, 0.5 μM of each primer and 0.25 μM of each probe were used. All quantitative reverse-transcription PCRs were performed in triplicates using the SuperScript® III Platinum® One-Step qRT-PCR kit (Invitrogen/Life Technologies, Darmstadt, Germany) according to the manufacturer’s instructions. Experiments were performed on a Stratagene Mx3005p (Agilent Technologies, Waldbronn, Germany) with 30 min at 50°C and 2 min at 95°C followed by 40 cycles of 15s at 95°C and 30s at 60°C. The lengths of the amplicons detected by the RACGAP1, TOP2A, Ki67 and CALM2 assays were 86bp, 104bp, 108bp and 72bp, respectively, with PCR efficiencies [E = 1^(10-slope)^] of 101.1%, 98.7%, 97.8% and 99.7%, respectively. Samples were considered eligible for further investigation (N = 875, Fig 1) when the cycle threshold (CT) values of the housekeeping gene were ≤33.5 (triplicate mean values). Relative expression levels (relative quantification, RQ) of the target transcripts were calculated as 40–DCT values (DCT = mean CT target gene–mean CT housekeeping gene) to yield positively correlated numbers and to facilitate comparisons [44]. A commercially available human reference RNA (Stratagene qPCR Human Reference Total RNA, Agilent Technologies, Waldbronn, Germany) was used as positive control. No-template controls were assessed in parallel to exclude contamination.

### Score determination

Regarding the risk for recurrence score (RRS), three proliferation markers were examined: RACGAP1, TOP2A and Ki67. The optimal RRS, in terms of disease-free survival (DFS) and overall survival (OS), was based on the analyses of co-expression of these genes.

The Adjuvant! Online score (AOS) provided a 10-year estimate of relapse and mortality risk in breast cancer patients treated with adjuvant systemic therapy, as previously published in detail [19, 45].

The modified clinical treatment score (hereafter CTS) integrated the prognostic information from age, nodal status, tumor size, histological grade and treatment, as previously published [46], with slight modifications to fit our cohort, since it had significant differences in tumor and patient characteristics. It should be noted that our cohort had only 4 node-negative patients, which were included together with the 1–3 positive nodes group when the CTS was calculated. In addition, all of our patients were high-risk according to St. Gallen criteria and all received chemotherapy in the adjuvant setting (one of three different treatment arms), which are the treatment arms taken into account when calculating the CTS.

A multivariate Cox proportional hazards regression model with the above factors as independent variables was run in terms of DFS and OS. The predicted values were estimated for each observed time and the 10-year estimates were used. The formula for the CTS model regarding the presentation of the coefficients used for OS is as follows: (-1.134) * nodes_0–3 + (-0.434) * size_≤2 cm + (-0.168) * grade_I-II + (0.006) * age + (-0.354) * HE1000-E-T-CMF arm + (-0.030) * HE1000-ET-CMF arm + (-0.167) * HE1097-E-T-CMF arm. The formula for DFS is: (-0.871) * nodes_0–3 + (-0.368) * size_≤2 cm + (-0.049) * grade_I-II + (0.002) * age + (-0.219) * HE1000-E-T-CMF arm + (-0.022) * HE1000-ET-CMF arm + (-0.041) * HE1097-E-T-CMF arm.

### Statistical methodology

Categorical variables were presented as frequencies and percentages, while continuous as means with standard deviation (SD), median and range. Associations among the three scores were examined using the Pearson’s correlation coefficient and ROC curve analysis, where appropriate.

DFS was measured from the time of diagnosis until verified disease progression, death or last contact, whichever occurred first, while OS from diagnosis until death from any cause or date of last contact. Time-to-event distributions were estimated using Kaplan-Meier curves. Cox proportional hazards models and log-rank tests were used to examine the prognostic significance for OS and DFS.

To assess the overall discriminative ability of RRS or AOS vs. CTS we used two approaches: a) changes in the likelihood ratio (LR) values (LR-Δχ^2^), which provide an estimate of the relative increase in the amount of information when either RRS or AOS is added to CTS; and b) the concordance index (C-index), a measure of concordance for time-to-event data. Increasing C-index values from 0.5 to 1.0 indicate improved prediction. More specifically, the C-index is defined as the probability that a subject from the event group has a higher predicted probability of having an event than a subject from the non-event group.

These two approaches are used in parallel since the C-index, even though being directly interpretable, is not sensitive for detecting small differences between models, as it is based on ranks. In contrast, the LR test is a more sensitive statistic but it requires modeling assumptions to be satisfied.

Time-dependent receiver operating characteristic (ROC) curve analysis was done in order to assess the discriminative ability of the models across time and not just for the 10-year time point estimates [47, 48]. Three estimators were used and compared: the nearest neighbor estimator (NNE), the Kaplan-Meier (KM) and the incidence/dynamic estimator.

To further investigate calibration (bias between actual and predicted outcome), continuous scores (CTS and AOS) were categorized into 3 risk classes based on the predicted 10-year risk, namely, low (<10%) vs. medium (10%-20%) vs. high (>20%) for DFS and low (<20%) vs. medium (20%-40%) vs. high (>40%) for OS. The risk class comparisons among all three scores were presented as bar charts for the 10-year estimates and Kaplan-Meier curves for the total follow-up period. Regarding the RRS score, since the co-expression variable that used two of the three genes was binary, the score was binary as well, classifying patients into two risk classes: low-risk (at least one gene with low mRNA expression) vs. high-risk (both genes with high mRNA expression).

In order to assess the general fit and prognostic ability of the CTS score, a sample splitting method was adopted. More specifically, the dataset was split in two parts, a training set and a validation set. The CTS model was created in the training set, while its prognostic ability was evaluated in the validation set. This procedure was performed on 100 random splits and mean results were presented.

All univariate tests were two-sided and significance level was set at α = 0.05. The analysis was conducted in the whole cohort of patients, as well as in the groups defined by IHC subtypes, HER2 status and nodal status (number of positive nodes). The results of this study are presented according to reporting recommendations for tumor marker prognostic studies [30]. No adjustments for multiple comparisons were made. The SAS software was used for statistical analysis (SAS for Windows, version 9.3, SAS Institute Inc., Cary, NC).

## Results

### Basic patient and clinical characteristics

In total 875 patients were included in the analysis. Basic patient and tumor characteristics, as well as their distribution by IHC subtypes are shown in S2 Table. Patients were classified to breast cancer subtypes defined by immunohistochemistry as follows: Luminal A (ER-positive and/or PgR-positive, HER2-negative, Ki67^low^); Luminal B (ER-positive and/or PgR-positive, HER2-negative, Ki67^high^); Luminal-HER2 (ER-positive and/or PgR-positive, HER2-positive); HER2-enriched (ER-negative, PgR-negative, HER2-positive); and triple-negative breast cancer (TNBC) (ER-negative, PgR-negative, HER2-negative).

Six hundred twenty-one patients (71.0%) underwent modified radical mastectomy, while 676 (77.2%) have been initially diagnosed with invasive ductal carcinoma. The majority of the patients have had >3 positive nodes removed (59.8%) and had tumors >2 cm in size (70.0%). Seventy-eight percent of the patients were ER/PgR-positive, while 84.2% had received taxanes, 78.8% adjuvant hormonotherapy and 74.8% adjuvant radiotherapy.

At a median follow-up time of 106.1 months (range 0.1–166.7), 232 deaths (26.5%) and 316 relapses (36.1%) had occurred.

### RRS score calculation

Three proliferation markers were examined in this project: RACGAP1, TOP2A and Ki67 mRNA expression. The distribution of continuous 40-DCT values for all three mRNA expression markers is shown in S1 Fig. For each marker, the three quartile cut-offs were examined as possible thresholds for prognostic significance in terms of DFS and OS, since no natural cut-offs were detected for any of the three markers. Regarding DFS, significance was not reached for any marker cut-off. On the contrary, the 1^st^ quartile, the 3^rd^ quartile and the median (25%, 75% and 50% percentiles) were significantly associated with OS for RACGAP1, TOP2A and Ki67, respectively. Poor OS was associated with high RACGAP1, TOP2A and Ki67 mRNA expression (S3 Table), while in multivariate analysis only TOP2A retained significance for OS (HR = 1.57, 95% CI 1.15–2.14, Wald’s p = 0.005). In an effort to improve prognostic significance, combinations of the mRNA expression of the genes were tested. When all three markers were assessed for combined prognostic ability, Ki67 retained its significance only when it was combined with RACGAP1, resulting to an unfavorable prognostic value for the co-expression (both with high expression vs. at least one low) (HR = 1.32, 95% CI 1.02–1.71, Wald’s p = 0.034, log-rank p = 0.034), while when combined with TOP2A the prognostic value for the co-expression was marginally significant (HR = 1.31, 95% CI 0.95–1.80, Wald’s p = 0.096, log-rank p = 0.095) (S3 Table). In addition, RACGAP1 and TOP2A mRNA co-expression was an adverse prognostic factor for OS (HR = 1.38, 95% CI 1.03–1.85, Wald’s p = 0.032, log-rank p = 0.031) (S2 Fig).

In multivariate analysis, only the combination of high RACGAP1 and TOP2A mRNA co-expression (both with high expression vs. at least one low) was associated with worse outcome for OS (HR = 1.52, 95% CI 1.10–2.09, Wald’s p = 0.011). Given the above results, the RRS score was based on the co-expression of RACGAP1 and TOP2A.

### AOS and RRS scores

**Fig 2:**
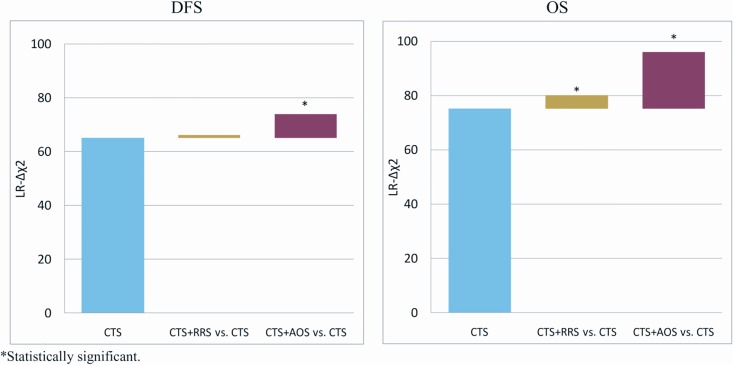
Prognostic information of CTS and improved significance when adding RRS or AOS to CTS. Changes in the likelihood ratio (LR) values (LR-Δχ^2^) are shown in the y-axis for disease-free survival (left panel) and overall survival (right panel).

Concerning the whole study cohort, AOS added significant prognostic information to CTS for both DFS and OS (LR-Δχ^2^ 8.7, p = 0.003 and LR-Δχ^2^ 20.8, p<0.001, respectively), while RRS only in terms of OS (LR-Δχ^2^ 4.8, p = 0.028) (Fig 2 and Tables 2 and 3).

**Table 2:** Prognostic information among models used in terms of DFS.

DFS	N of patients	N of events	CTS (8 df)		RRS (1 df)		AOS (1 df)		CTS+RRS (9 df)		CTS+AOS (9 df)		CTS+RRS vs. CTS (1 df)		CTS+AOS vs. CTS (1 df)	
			LR-Δχ^2^	p	LR-Δχ^2^	p	LR-Δχ^2^	p	LR-Δχ^2^	p	LR-Δχ^2^	p	LR-Δχ^2^	P	LR-Δχ^2^	p
All patients	875	316	65.1	<0.001	0.8	0.38	53.2	0.14	66.2	<0.001	73.8	<0.001	1.1	0.29	8.7	0.003
By IHC subtype																
Luminal A	201	55	12.6	0.083	1.6	0.21	10.4	0.001	14.8	0.062	17.4	0.026	2.3	0.13	4.8	0.028
Luminal B	313	118	32.2	<0.001	0.6	0.42	16.0	<0.001	33.6	<0.001	37.0	<0.001	1.3	0.24	4.8	0.029
Luminal-HER2	109	45	10.8	0.15	0.5	0.48	9.2	0.002	10.9	0.21	12.3	0.14	0.1	0.81	1.5	0.23
HER2-Enriched	87	31	11.1	0.14	<0.1	0.83	1.6	0.20	11.4	0.18	11.1	0.20	0.4	0.55	<0.1	0.94
Triple-negative	96	43	7.6	0.37	<0.1	0.83	7.1	0.008	7.6	0.47	9.8	0.28	<0.1	>0.99	2.2	0.14
By HER2 status																
HER2-positive	196	76	16.5	0.021	0.4	0.53	10.0	0.002	16.5	0.036	17.6	0.025	<0.1	0.98	1.0	0.31
HER2-negative	621	220	46.3	<0.001	3.2	0.072	37.7	<0.001	49.8	<0.001	56.5	<0.001	3.5	0.060	10.2	0.001
By nodal status (-1 df for CTS)																
0–3	351	75	5.6	0.47	0.8	0.38	2.4	0.12	6.1	0.53	5.8	0.56	0.5	0.50	0.2	0.64
>3	524	241	7.6	0.27	0.5	0.50	12.3	0.001	8.3	0.31	16.9	0.018	0.7	0.39	9.3	0.002

Patients were classified to breast cancer subtypes defined by immunohistochemistry as follows: Luminal A (ER-positive and/or PgR-positive, HER2-negative, Ki67^low^); Luminal B (ER-positive and/or PgR-positive, HER2-negative, Ki67^high^); Luminal-HER2 (ER-positive and/or PgR-positive, HER2-positive); HER2-enriched (ER-negative, PgR-negative, HER2-positive); and triple-negative breast cancer (TNBC) (ER-negative, PgR-negative, HER2-negative).

DFS, disease-free survival; N, number; CTS, clinical treatment score; RRS, risk for recurrence score; AOS, adjuvant online score IHC, immunohistochemistry.

**Table 3:** Prognostic information among models used in terms of OS.

OS	N of patients	N of events	CTS (8 df)		RRS (1 df)		AOS (1 df)		CTS+RRS (9 df)		CTS+AOS (9 df)		CTS+RRS vs. CTS (1 df)		CTS+AOS vs. CTS (1 df)	
			LR-Δχ^2^	p	LR-Δχ^2^	p	LR-Δχ^2^	p	LR-Δχ^2^	p	LR-Δχ^2^	p	LR-Δχ^2^	p	LR-Δχ^2^	p
All patients	875	232	75.1	<0.001	4.4	0.036	75.3	<0.001	79.9	<0.001	96.0	<0.001	4.8	0.028	20.8	<0.001
By IHC subtype																
Luminal A	201	32	19.1	0.008	4.5	0.034	10.7	0.001	25.2	0.002	26.3	0.001	6.1	0.013	7.2	0.007
Luminal B	313	86	31.0	<0.001	3.7	0.056	27.1	<0.001	35.9	<0.001	39.3	<0.001	4.8	0.028	8.3	0.004
Luminal-HER2	109	34	14.7	0.040	0.5	0.50	12.1	0.001	14.7	0.065	19.1	0.014	<0.1	0.91	4.4	0.035
HER2-Enriched	87	20	11.6	0.11	0.1	0.73	3.0	0.081	11.9	0.15	11.7	0.17	0.3	0.59	<0.1	0.90
Triple-negative	96	39	10.2	0.18	0.3	0.58	7.4	0.007	10.8	0.21	12.1	0.15	0.6	0.44	1.9	0.17
By HER2 status																
HER2-positive	196	54	23.3	0.002	0.3	0.58	11.0	0.001	23.3	<0.001	24.8	0.002	<0.1	0.85	1.5	0.22
HER2-negative	621	161	53.3	<0.001	9.7	0.002	61.3	<0.001	61.9	<0.001	76.7	<0.001	8.7	0.003	23.4	<0.001
By nodal status (-1 df for CTS)																
0–3	351	43	11.8	0.067	3.7	0.053	3.3	0.069	14.4	0.045	11.8	0.11	2.6	0.11	<0.1	0.91
>3	524	189	11.1	0.085	2.4	0.12	24.7	<0.001	13.8	0.054	35.0	<0.001	2.7	0.10	23.9	<0.001

For details on patient classification to breast cancer subtypes see footnote legend to Table 2.

OS, overall survival; N, number; CTS, clinical treatment score; RRS, risk for recurrence score; AOS, adjuvant online score; IHC, immunohistochemistry.

Subgroup analysis showed that AOS in addition to CTS added prognostic information in Luminal A (LR-Δχ^2^ 4.8, p = 0.028), Luminal B (LR-Δχ^2^ 4.8, p = 0.029), HER2-negative patients (LR-Δχ^2^ 10.2, p = 0.001) and patients with >3 positive nodes (LR-Δχ^2^ 9.3, p = 0.002) in terms of DFS (Table 2). On the contrary, RRS added prognostic information to CTS in HER2-negative patients only (LR-Δχ^2^ 3.5, p = 0.060).

In terms of OS, AOS added prognostic information to CTS in Luminal A (LR-Δχ^2^ 7.2, p = 0.007), Luminal B (LR-Δχ^2^ 8.3, p = 0.004), HER2-negative patients (LR-Δχ^2^ 23.4, p<0.001) and patients with >3 positive nodes (LR-Δχ^2^ 23.9, p<0.001). RRS did not added prognostic information in the patients with >3 positive nodes, but did in Luminal A (LR-Δχ^2^ 6.1, p = 0.013), Luminal B (LR-Δχ^2^ 4.8, p = 0.028) and HER2-negative patients (LR-Δχ^2^ 8.7, p = 0.003) (Table 3).

Moreover, every other possible combination of scores and the corresponding comparisons are presented in S4 and S5 Tables, in order to enrich the presentation of the scores’ prognostic value. The patients suitable for using CTS combined either with RRS, AOS or both RRS and AOS (Tables 2 and 3, S4 and S5 Tables) are Luminal A and B patients, HER2-negative patients and those with >3 positive nodes. However, significant differences regarding added prognostic significance are observed when RRS is used alone in comparison with the combinations with the other two scores, indicating that RRS is important when used in combination with the other scores.

**Fig 3:**
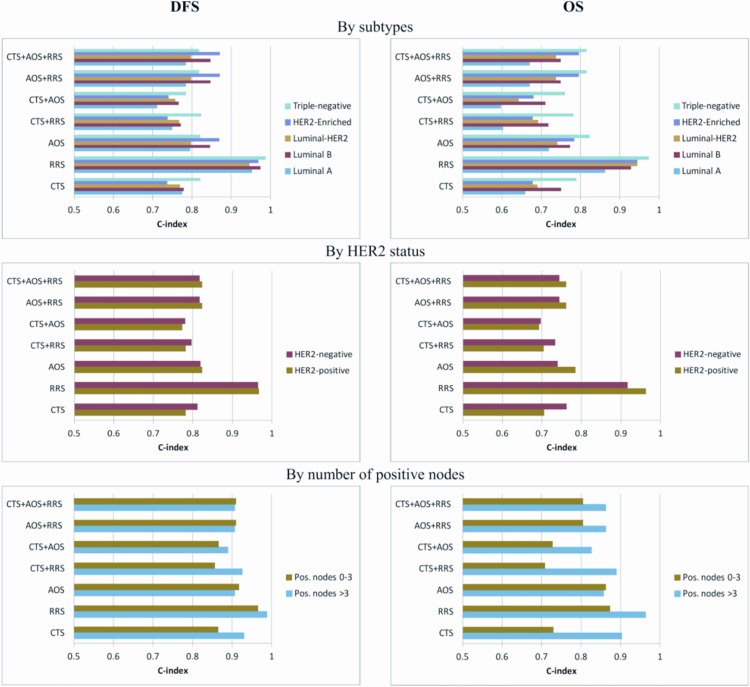
Discrimination power by the concordance index (C-index) for the three scores alone and their combinations. C-indices by IHC subtypes, HER2 status and number of positive nodes are shown in the x-axis for disease-free survival (left panels) and overall survival (right panels).

C-index results by IHC subtypes, HER2 status and number of positive nodes are presented in Fig 3, regarding the three scores alone and their combinations. All scores and their combinations were informative in terms of the C-index (all >0.6). The higher C-indices were estimated for the subgroups of triple-negative patients and patients with >3 positive nodes, in the case of both DFS and OS for each score model. Regarding score comparisons, the higher values of C-indices were observed for the RRS score in every subgroup. This implies that RRS provides significant prognostic information, which however is not available when used on top of other scores.

Time-dependent ROC curve analyses for DFS (S3 Fig) and OS (S4 Fig) showed that for all scores and their combinations, a peak in the area under the curve (AUC) values estimated by the NNE and KM methods is seen in the first 2 to 3 years, which however is lost at the 10-year time point, with RRS having the highest AUC values.

### CTS score validation

A sample-splitting approach was used in order to create and validate the CTS, as well as the additive prognostic information derived from AOS. The sample was split into two parts, a training and a validation set. The first was used to create the CTS score, while the second to assess the prognostic ability of the created CTS. This procedure was replicated 100 times and the average changes in the likelihood ratio (LR) values are presented in S6 and S7 Tables in terms of DFS and OS, respectively. The percentages of reaching significance at the 1% level are also presented.

In terms of DFS, the results indicated that addition in the prognostic significance is not very stable. In 73 out of the 100 replicates, the addition of AOS to CTS was significant at the 5% level. The corresponding percentages for the Luminal B and HER2-negative patients are 74% and 71%, respectively, while for the >3 positive nodes patients the percentage was 76%.

Regarding OS in the whole sample, the addition of AOS to CTS was significant at α = 5% for 95 of the 100 replicates. Very similar results occurred in HER2-negative and Luminal B patients, 94% and 96% respectively, while in HER2-positive patients only 23 of the 100 replicates showed significance at α = 5%. Ninety-five of the 100 replicates showed significance in the subgroup of patients with >3 positive nodes. Of note, considering the 1% level of significance, the above results in the mentioned subgroups (whole sample, HER2-negative, Luminal B and >3 positive nodes) showed stability, with the percentages ranging from 80% to 88%.

### Comparison of predicted risk for relapse with CTS, RRS and AOS

**Fig 4:**
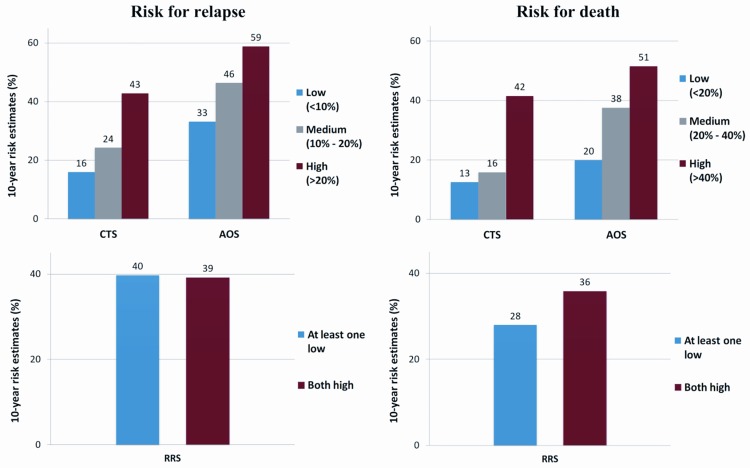
Ten-year risk estimates for CTS, AOS and RRS according to risk class categories. Risk for relapse is shown in the left panels and risk for death in the right panels, according to low, medium and high CTS and AOS. For RRS, two risk classes were available, with the red bars representing the risk for patients with high mRNA expression for both RACGAP1 and TOP2A and the blue bars when at least one of the genes had low mRNA expression (numbers on bars: percent of 10-year risk estimate).

**Fig 5:**
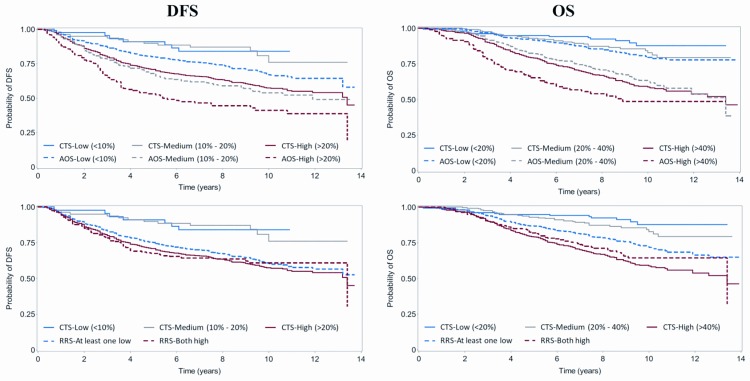
Kaplan-Meier curves for CTS, RRS and AOS according to risk class categories. Predicted (estimated) probabilities are shown for disease-free survival in the left panels and for overall survival in the right panels.

To illustrate the prognostic power of the scores, the 10-year risk estimates were compared within risk classes (Fig 4). AOS always provided higher 10-year risk estimates compared to CTS and moreover the relative differences between the estimates of the high- vs. low-risk groups were smaller than CTS (DFS: 169% vs. 79% and OS: 223% vs. 155% for CTS and AOS, respectively). On the other hand, as noted above, RRS failed to discriminate the 10-year risk for relapse, while regarding 10-year risk for death the relative difference between the estimates of the both high vs. at least one low risk groups was 29% (Fig 4). However, the combined relationship of CTS and AOS shows that maximum differentiation can be achieved between the low risk group defined by the CTS and the high risk group defined by the AOS for both DFS and OS (Fig 5).

Further assessment was performed by comparing the predicted (estimated) vs. observed DFS and OS probabilities of CTS, AOS added to CTS and RRS added to CTS within well-established prognostic factor subgroups, such as age, positive nodes, tumor size and histological grade.

**Fig 6:**
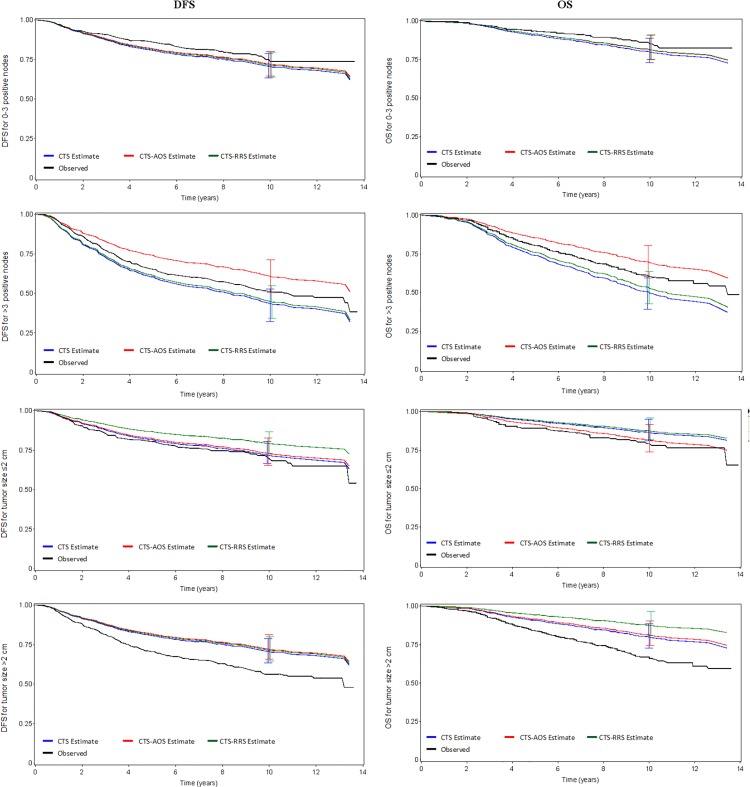
Observed versus predicted (estimated) DFS and OS probabilities for CTS, CTS+RRS and CTS+AOS. Disease-free survival probabilities are shown in the left panels and overall survival probabilities in the right panels, according to the number of positive nodes (first four panels) and tumor size (last four panels). Vertical bars depict 95% confidence intervals of the predicted outcome at the 10-year time point.

Across all models calibration was low implying that the models when assessed within sub-cohorts defined by the parameters used to calculate CTS, a loss in predictive accuracy is observed, since the respective parameter is “neutralized”. In the subgroup of patients with >3 positive nodes, the addition of AOS to CTS differentiated predicted risk for both DFS and OS (Fig 6). The addition of AOS to CTS also differentiated the prediction in patients with histological grade I-II in terms of OS (S5 Fig).

Slight differentiations were also identified when RRS was added to CTS in terms of OS in the subgroup of patients with >2 cm tumor size, while in terms of DFS in the subgroup of patients with tumor size ≤2 cm. Nevertheless, these differences do not correspond with predictive accuracy, considering the under- and over-estimation of the observed DFS and OS probabilities (Fig 6).

### Concordance between CTS, RRS and AOS

Correlation analysis found CTS to correlate with the AOS estimates, while strong correlations were estimated for the Luminal-HER2 patients (r = 0.71 for both DFS and OS), 0–3 positive nodes patients (r = 0.73 for DFS and r = 0.72 for OS) and >3 positive nodes patients (r = 0.43 for DFS and r = 0.52 for OS) (S8 and S9 Tables).

ROC curve analysis showed that RRS distinction was not consistent with the estimations of relapse and overall mortality derived by AOS and CTS. The cases in which an association between RRS and relapse might exist were in HER2-Enriched patients and those with >3 positive nodes, with values for area under the curve (AUC) 0.667 and 0.565, respectively (S10 Table). In the case of overall mortality, Luminal A, HER2-Enriched, HER2-positive and patients with 0–3 positive nodes had AUC values slightly large (0.622, 0.674, 0.624 and 0.603, respectively) (S11 Table).

Lastly, the concordance of CTS, AOS and RRS, in all risk classes, is summarized in S12 Table. In the case of CTS and AOS, CTS categorized more patients as high-risk and AOS more patients as low-risk for either DFS or OS. As a result the majority of the patients classified as “high-risk” by the AOS were also classified as “high-risk” by the CTS, but the reverse was not true, while almost all the CTS low-risk patients were also low-risk according to AOS. The number of patients categorized as low-risk by either CTS or AOS were fairly similar to the RRS low-risk class (at least one mRNA gene expression low), but those categorized as high-risk by the RRS (both genes high) were not similar to the patients categorized as high-risk by either CTS or AOS.

## Discussion

In the present study, we developed a risk for recurrence score (RRS), based on mRNA expression of two proliferation markers (RACGAP1 and TOP2A), in high-risk early breast cancer patients and evaluated its ability to predict risk for relapse and death. In addition the Adjuvant! Online score (AOS) was also determined for each patient. We then examined whether the RRS and/or AOS, added to CTS, provided more prognostic information in terms of DFS or OS, in much the same way Dowsett et al. [8] evaluated the ability of RS and ROR to improve the prognostic information of the CTS. The CTS was slightly modified from what was previously published [46], in order to fit our cohort, since it had significant differences in tumor and patient characteristics. It should be noted that our cohort comprised of high-risk early breast cancer patients that were all treated with chemotherapy (one of three regimens) and that, with regard to ER/PgR and HER2 status, both negative and positive patients were included in our study.

In our cohort of 875 high-risk patients, AOS added significant prognostic information to CTS for both DFS and OS, while RRS only in terms of OS, as detected by changes in the likelihood ratio (LR) values. When both RRS and AOS were added to CTS, a strong improvement in prognostic information was observed for both DFS and OS, indicating that RRS is important when used in combination with the other two scores. The clinical significance of our findings is that AOS, when used together with the CTS, adds significant prognostic information in terms of both DFS and OS.

It appears that by combining AOS with CTS, maximum differentiation can be achieved between the low-risk group defined by the CTS and the high-risk group defined by the AOS for both DFS and OS. This might prove to be of importance in identifying patients that might derive benefit from novel, possibly more aggressive adjuvant treatments in the future, since all of our patients received adjuvant chemotherapy, as opposed to the Dowsett et al. patients [8] that were postmenopausal women treated with hormonal therapy. It should be noted however, that, as indicated in the first report of the ATAC randomized trial [49], approximately 21% of the Dowsett et al. patients also received chemotherapy after primary surgery. Nevertheless, if our findings are validated in an independent cohort, the combined use of the two scores might prove to be an important and powerful tool in determining the prognosis of high-risk early breast cancer patients.

In the Dowsett et al. study [8], the RS and ROR, derived from the Oncotype DX and PAM50, respectively, were compared with the IHC4 score, a prognostic model that combines quantitative IHC parameters, such as ER, PgR, HER2 and Ki67. In this study, clinical subtyping, as defined by the IHC parameters of the IHC4 score, was used as a stratification factor for the subgroup analysis rather than being added to the CTS and compared to the other scores. The main reason behind this strategy is that each different breast cancer subtype represents a different entity having unique prognosis and it was therefore deemed important to evaluate the prognostic value of the scores in subgroups of patients according to immunohistochemical sybtype.

Prognostic information was improved in terms of DFS and OS, as detected by changes in the LR values, in the Luminal A and B subtypes, HER2-negative patients and patients with more than 3 positive nodes, when AOS was added to CTS. By adding RRS to CTS, DFS was improved in HER2-negative patients and OS in Luminal A and B subtypes and HER2-negative patients. There was also a sufficient discrimination power for DFS and OS for both combinations in all breast cancer subtypes and all three scores were informative in terms of the C-index, with higher C-indices estimated in triple-negative patients and patients with more than 3 positive nodes. Of note RRS showed higher discrimination in all examined subgroups, which is however lost at longer follow-up periods, as time-dependent ROC curve analysis demonstrated. Additionally, AOS seemed to give higher risk estimates for relapse and mortality than CTS, while RRS failed to discriminate the 10-year risk for relapse. The addition of AOS to CTS differentiated the prediction for OS and DFS in patients with more than 3 positive nodes and in those with grade I-II tumors and for OS in patients with tumor size ≤2 cm. There was also a strong correlation in prediction among AOS and CTS, especially in Luminal-HER2 patients and patients with 0–3 positive nodes. However, RRS showed little consistency in risk for relapse and death prediction compared with AOS and CTS.

When the three risk scores were examined for possible concordance, moderate associations were identified, especially in case of CTS and RRS. That was expected, since the scores had similarities, but significant differences, as well. The patients most suitable for using CTS combined with RRS, AOS or both RRS and AOS are Luminal A and B patients, HER2-negative patients and those with >3 positive nodes. This is in agreement with the findings of a recent study by Sestak et al. [50], where patients previously enrolled in the TransATAC and ABCSG8 randomized trials were assessed in terms of distant recurrence in the period following 5 years from diagnosis. The PAM50-based ROR offered significant value in predicting prognosis when added to the CTS, beyond that provided by CTS in node-negative/HER2-negative patients.

Different groups have evaluated prognostic indices and Adjuvant! Online predictions in the past. Breast cancer index (BCI) is a predictor of outcome, which combines two independent biomarkers assessing estrogen-mediated signaling and tumor grade. In a study by Jankowitz et al. [51], BCI was found to have additive utility to Adjuvant! Online. The combination of BCI and Adjuvant! Online increased the predictive accuracy for risk for recurrence from 66% to 76% in all patients and from 65% to 81% in the tamoxifen-only treated group. Another study by Hearne et al. [52] compared the Nottingham prognostic index (NPI), which combines nodal status, tumor size and histological grade in a simple formula, with Adjuvant! Online in young (<40 years old) breast cancer patients and found a strong correlation between the 10-year predicted survival by NPI and Adjuvant! Online, which were very similar to the actual survival of the patients. However, in two earlier studies [20, 21] Adjuvant! Online was found to significantly overestimate overall survival and event-free survival, resulting in the underestimation of breast cancer mortality. More recently, the Dutch group by van Kleef et al. [24], also identified that the Adjuvant! Online score does not accurately predict breast cancer specific survival and event-free survival in patients with early breast cancer, evidence that strengthens our data, by revealing the weaknesses of the above score, when used alone, in accurately assessing prognosis. However, even in our study, the combination of AOS with CTS appears to overestimate DFS and OS in patients with more than 3 positive nodes and patients with tumor size >2 cm. The same appears to be true when RRS is combined to CTS, not only in patients with tumor size >2 cm, but also in patients with smaller tumors.

It appears therefore, based on our findings, that combining AOS with CTS is better in predicting DFS versus either score alone in a statistically significant manner, while prediction of OS was better when combining CTS with either AOS or RRS compared to either score alone. This is in agreement with a recent study by Zhao et al. [9] aiming to validate the prognostic power of multiple gene signatures versus histopathological characteristics, such as ER status, which showed that the prognostic power of gene signatures when used alone is limited.

Summarizing, the current study has shown a clear benefit in predicting overall survival of high-risk early breast cancer patients when combining the score based on clinicopathological parameters (CTS) with either the score based on Adjuvant! Online (AOS) or the score based on our proliferation gene signature (RRS). According to subtypes, prognosis was improved in the Luminal A and Luminal B subtypes and in HER2-negative patients when AOS was added to CTS. These findings could possibly add on the information needed for the best risk prediction strategy in high-risk early breast cancer patients in a rather simple and inexpensive way, considering the added value of the new gene expression signatures and their cost. Therefore, in everyday clinical practice, and especially when practicing in countries where resources are limited, our study indicates that prediction value in high-risk early breast cancer patients, especially in Luminal A and Luminal B subtypes, HER2-negative patients and those with >3 positive nodes, significantly improves with the combination of three rather easy to perform scores.

## Conclusions

This study adds on the already existing knowledge that there is no single accurate prediction tool for all breast cancer subtypes. Novel biomarkers are required to assist clinicians in breast cancer detection, risk stratification, treatment response and risk prognosis. Gene expression profiling has the potential of improving breast cancer management. However, its clinical value cannot yet be claimed above and beyond the use of standard clinicopathological prognostic variables. The challenge is the evaluation of the contribution of both molecular and clinical data in predicting outcome in a prospective manner. Therefore, developing integrative models of clinical and molecular factors will increase our understanding of breast cancer and accelerate the progress towards tailored medicine. The findings of the present study could be further evaluated in a prospective manner, possibly integrating in a future study the AOS, RRS and CTS, as well as the new gene expression signatures in an attempt to evaluate their utility and cost effectiveness. The ultimate goal would be to gradually move from utilizing expensive empirical oncology treatments to tailored approaches, aiming to have a valuable clinical benefit taking into account healthcare economics at the same time.

## Supporting Information

S1 Fig Distribution of continuous 40-DCT values for all three mRNA expression markers. (TIF)

S2 Fig Kaplan-Meier curves for DFS and OS according to RACGAP1 and TOP2A mRNA co-expression. (TIF)

S3 Fig Time-dependent ROC curve analysis for disease-free survival, with area under the curve (AUC) value plots. AUC value plots for 10-year (120-month) estimates and AUC estimates across time are presented, using three estimators: nearest neighbor estimator (NNE), Kaplan-Meier (KM) and the incidence/dynamic estimator. Multiple four-panel ROC curves are presented for each the three scores (CTS, AOS and RRS) and their combinations. FP, false positive; TP, true positive.
(TIF)

S4 Fig Time-dependent ROC curve analysis for overall survival, with area under the curve (AUC) value plots. For details see S3 Fig legend.
(TIF)

S5 Fig Observed versus predicted (estimated) DFS and OS probabilities for CTS, CTS+RRS and CTS+AOS. Disease-free survival probabilities are shown in the left panels and overall survival probabilities in the right panels, according to histological grade (first four panels) and age (last four panels). Vertical bars depict 95% confidence intervals of the predicted outcome at the 10-year time point.
(TIF)

S1 Table Clinical trial characteristics and treatment regimens administered. (TIF)

S2 Table Basic patient and tumor characteristics by immunohistochemical (IHC) subtypes. (TIF)

S3 Table Univariate analysis for DFS and OS of the three mRNA markers and their co-expression. All three cut-offs were analyzed, with only the significant OS cut-offs used for the co-expression analysis.
(TIF)

S4 Table Prognostic information among models used, in terms of disease-free survival (DFS), for every combination. (TIF)

S5 Table Prognostic information among models used, in terms of overall survival (OS), for every combination. (TIF)

S6 Table Prognostic information among models used, in terms of DFS, by sample splitting (validation dataset results). (TIF)

S7 Table Prognostic information among models used, in terms of OS, by sample splitting (validation dataset results). (TIF)

S8 Table Correlation between CTS predicted values in terms of DFS and relapse estimated by AOS. (TIF)

S9 Table Correlation between CTS predicted values in terms of OS and overall mortality estimated by AOS. (TIF)

S10 Table ROC curve analysis of RRS and AOS or CTS 10-year predicted values for DFS. Area under the curve values are presented.
(TIF)

S11 Table ROC curve analysis of RRS and AOS or CTS 10-year predicted values for OS. Area under the curve values are presented.
(TIF)

S12 Table Concordance of CTS, AOS and RRS, in all risk classes. Numbers of patients for every combination of risk classes are presented.
(TIF)
