# Supplementary figures and images for: Comparison of the Ability of Different Clinical Treatment Scores to Estimate Prognosis in High-Risk Early Breast Cancer Patients: A Hellenic Cooperative Oncology Group Study
Source: PLoS One. 2016 Oct 3;11(10):e0164013. doi: 10.1371/journal.pone.0164013 (PMC5047528; doi:10.1371/journal.pone.0164013)

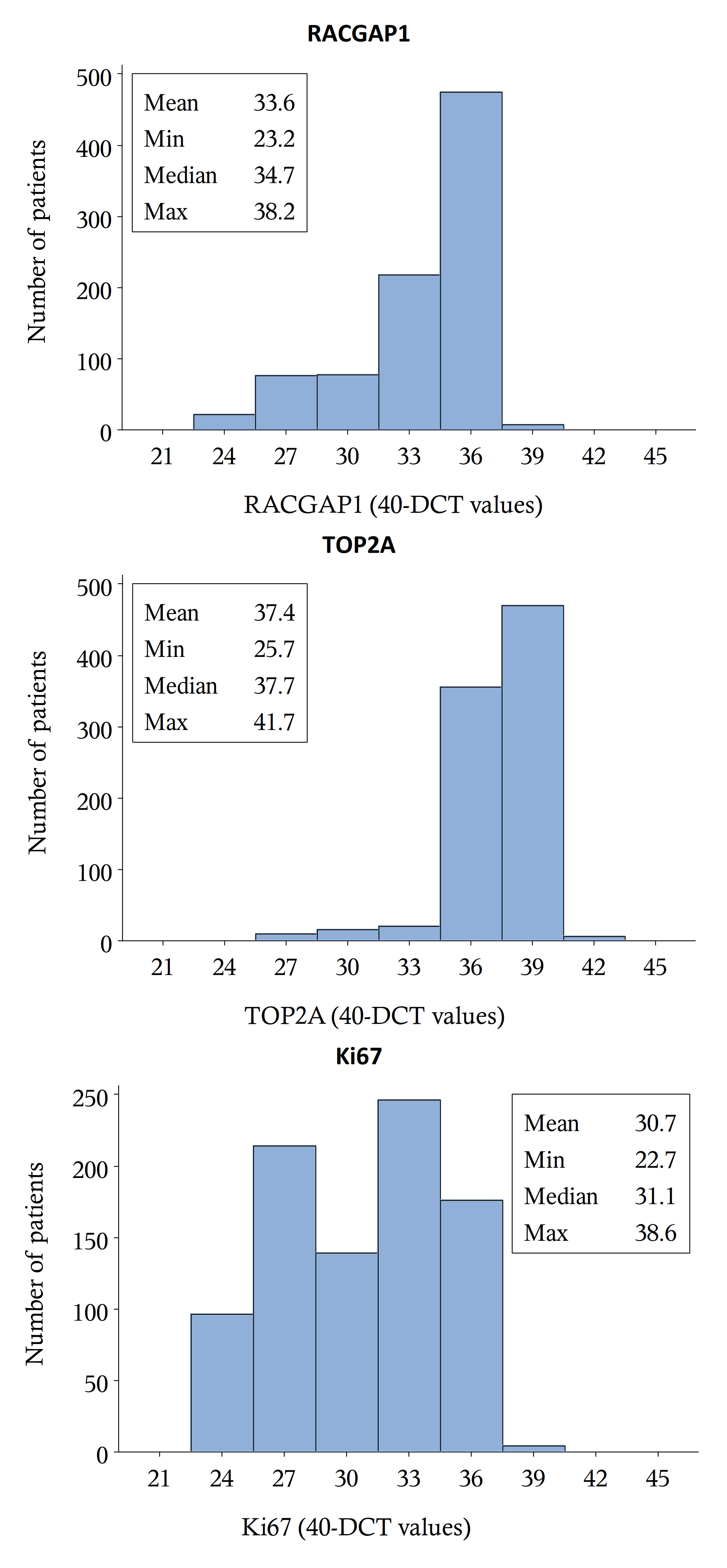

Supplement: S1 Fig — (TIF) [file pone.0164013.s001.tif]

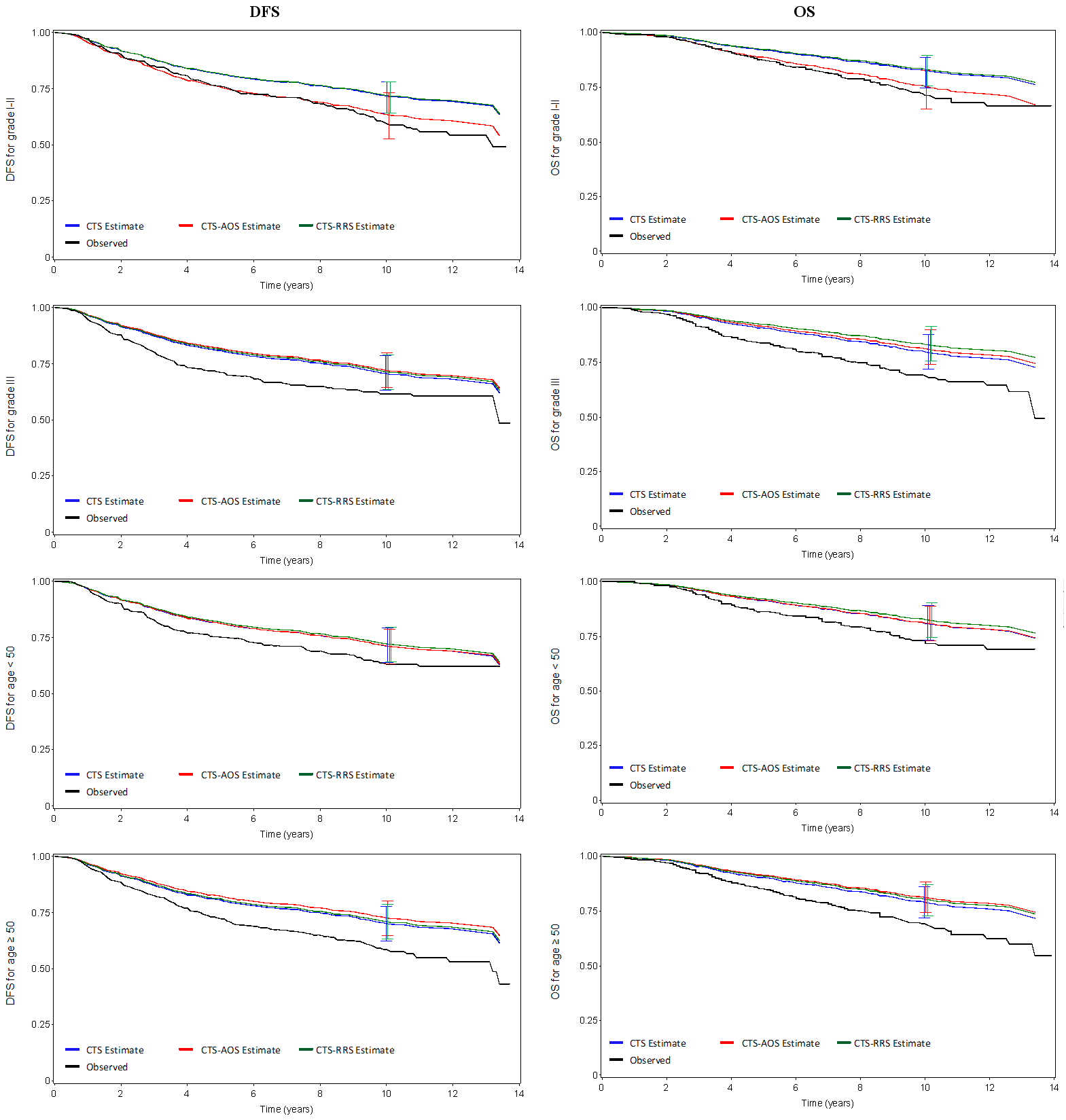

Supplement: S5 Fig — Disease-free survival probabilities are shown in the left panels and overall survival probabilities in the right panels, according to histological grade (first four panels) and age (last four panels). Vertical bars depict 95% confidence intervals of the predicted outcome at the 10-year time point. (TIF) [file pone.0164013.s005.tif]
